# From beech wood to itaconic acid: case study on biorefinery process integration

**DOI:** 10.1186/s13068-018-1273-y

**Published:** 2018-10-11

**Authors:** Lars Regestein, Tobias Klement, Philipp Grande, Dirk Kreyenschulte, Benedikt Heyman, Tim Maßmann, Armin Eggert, Robert Sengpiel, Yumei Wang, Nick Wierckx, Lars M. Blank, Antje Spiess, Walter Leitner, Carsten Bolm, Matthias Wessling, Andreas Jupke, Miriam Rosenbaum, Jochen Büchs

**Affiliations:** 1 AVT—Bio-chemical Engineering, RWTH Aachen University, Forckenbeckstr. 51, 52074 Aachen, Germany; 2 AVT—Chemical Process Engineering, RWTH Aachen University, Forckenbeckstr. 51, 52074 Aachen, Germany; 3 AVT—Fluid Process Engineering, RWTH Aachen University, Forckenbeckstr. 51, 52074 Aachen, Germany; 4 AVT—Enzyme Process Technology, RWTH Aachen University, Forckenbeckstr. 51, 52074 Aachen, Germany; 5 Institute of Technical and Macromolecular Chemistry, RWTH Aachen University, Worringerweg 2, 52064 Aachen, Germany; 6 Max Planck Institute for Chemical Energy Conversion, Stiftstraße 34-36, 45470 Mülheim an der Ruhr, Germany; 7 iAMB-Institute of Applied Microbiology, RWTH Aachen University, Worringerweg 1, 52064 Aachen, Germany; 8 Leibniz Institute for Natural Product Research and Infection Biology - Hans Knöll Institute, Adolf-Reichwein-Str. 23, 07745 Jena, Germany; 9 Center of Molecular Transformations, RWTH Aachen University, Worringerweg 1, 52074 Aachen, Germany; 10 Institut für Bioverfahrenstechnik, Technische Universität Braunschweig, Rebenring 56, 38106 Brunswick, Germany; 11 Institut für Organische Chemie, RWTH Aachen University, Landoltweg 1, 52074 Aachen, Germany; 12 Institut für Bio- und Geowissenschaften, Pflanzenwissenschaften (IBG-2), Forschungszentrum Jülich, Wilhelm-Johnen-Straße, 52425 Jülich, Germany

**Keywords:** Biorefinery process, Bio-chemical conversion, Bio-based platform chemical, Itaconic acid, Bioeconomy

## Abstract

Renewable raw materials in sustainable biorefinery processes pose new challenges to the manufacturing routes of platform chemicals. Beside the investigations of individual unit operations, the research on process chains, leading from plant biomass to the final products like lactic acid, succinic acid, and itaconic acid is increasing. This article presents a complete process chain from wooden biomass to the platform chemical itaconic acid. The process starts with the mechanical pretreatment of beech wood, which subsequently is subjected to chemo-catalytic biomass fractionation (OrganoCat) into three phases, which comprise cellulose pulp, aqueous hydrolyzed hemicellulose, and organic lignin solutions. Lignin is transferred to further chemical valorization. The aqueous phase containing oxalic acid as well as hemi-cellulosic sugars is treated by nanofiltration to recycle the acid catalyst back to the chemo-catalytic pretreatment and to concentrate the sugar hydrolysate. In a parallel step, the cellulose pulp is enzymatically hydrolyzed to yield glucose, which—together with the pentose-rich stream—can be used as a carbon source in the fermentation. The fermentation of the sugar fraction into itaconic acid can either be performed with the established fungi *Aspergillus terreus* or with *Ustilago maydis*. Both fermentation concepts were realized and evaluated. For purification, (in situ) filtration, (in situ) extraction, and crystallization were investigated. The presented comprehensive examination and discussion of the itaconate synthesis process—as a case study—demonstrates the impact of realistic process conditions on product yield, choice of whole cell catalyst, chemocatalysts and organic solvent system, operation mode, and, finally, the selection of a downstream concept.

## Background

The global change to renewable feedstocks drives the development of new processes for a wide range of functional platform chemicals and fuels [1–4]. An industrial production via biotechnological processes has to be adapted to new challenges such as recalcitrant raw materials, oxygenized molecules, and chemically highly demanding starting materials, such as lignocellulose [5]. Within the large spectrum of possible products, organic acids are particularly interesting as building blocks. Several studies have defined criteria for a competitive biotechnological process: Low price for bio-catalysts, flexible utilization of different (low price) feedstocks (straw, grass, wood, etc.), and low environmental impact [6–11]. The fact that optimized bioprocesses can already economically compete with their petrol-based counterparts is demonstrated by the strong market positions of citric and lactic acid, organic acids that are produced by fermentation in megaton scale [12]. For new processes, space–time yield and product titer have often to be drastically improved, especially for the bulk production of biofuels.

Beside the investigation of individual unit operations, there have been several research projects on process chains leading from plant biomass to the final products like lactic acid, succinic acid, and itaconic acid [13–16]. The development and optimization of (bio-)catalysts based on pure or idealized model substrates are absolutely necessary, but in case of biorefinery processes, it is doubtful whether the results can be transferred to an integrated process, where substrates contain impurities, or are only available in a diluted form.

Itaconic acid is considered as one of the most promising building blocks for the synthesis of a variety of product classes [2, 17]. It can be converted to the potential second-generation biofuel 3-methyltetrahydrofuran (3-MTHF), which has superior combustion and emission properties compared to gasoline [18]. Itaconic acid is currently produced at industrial scale by the filamentous fungus *Aspergillus terreus* from renewable substrates like molasses [19, 20]. However, *A. terreus* is sensitive to substrate impurities, which is reflected by the strong effect of several medium components on itaconic acid formation [20–25].

This study aims at analyzing the potential and pitfalls of the complete process chain for the conversion of plant biomass (beech wood) into the platform chemical itaconic acid, thereby identifying areas of future research needs.

### Conceptual overview of the itaconic acid process

A first general process scheme for production of itaconic acid from wooden biomass was already proposed by Kobayashi in 1978 [26]. After itaconic acid was mentioned as a very promising platform chemical [2], it became the focus of several larger research projects in the last decade. The “German Lignocellulose Feedstock Biorefinery Project”, investigated itaconic acid production from wood hydrolysates [27]. The project “Development of integrated production of polyitaconic acid from northeast hardwood biomass” investigated the utilization of hardwood, softwood, and corn gluten feed for the production of polyitaconic acid [28]. In both projects, inhibitory compounds negatively influenced the growth and itaconic acid production of *A. terreus*.

Since 2007, the German Cluster of Excellence “Tailor-Made Fuels from Biomass (TMFB)” aims at establishing innovative and sustainable processes for the conversion of plant biomass into fuels, for novel low-temperature combustion engines with high efficiency and low pollutant emission. In this project, itaconic acid is an intermediate platform chemical and can be converted into several potential fuel candidates, such as 3-methyltetrahydrofuran [29, 30]. The process can be classified as a “Lignocellulose Feedstock Biorefinery” according to the definition of Kamm and Kamm [31] and the German VDI [32], since it uses biomasses with low water content as raw materials, e.g., wood, straw, corn stover as well as cellulose-containing biomass and waste.

**Fig. 1:**
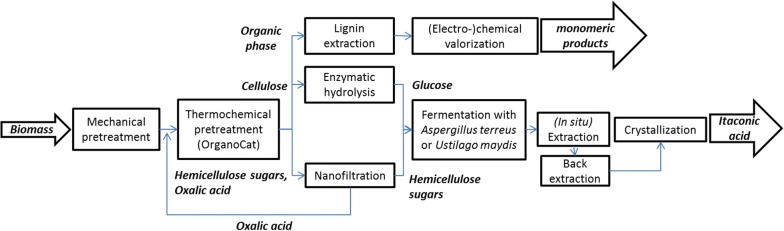
Block flow diagram from biomass to itaconic acid

The process chain from biomass to itaconic acid is shown, as block flow diagram in Fig. 1. The process starts with the mechanical pretreatment of beech wood [33, 34], which subsequently is subjected to chemo-catalytic biomass fractionation (OrganoCat) into three phases, which comprise cellulose pulp, aqueous hemicellulose, and organic lignin solutions [35–37]. Lignin is transferred to further chemical valorization [38–51]. The aqueous phase containing oxalic acid as well as hemi-cellulosic sugars (mainly pentoses) is treated by nanofiltration to recycle the catalyst back to the chemo-catalytic fractionation process and to concentrate the sugar hydrolysate. In a parallel step, the cellulose pulp is enzymatically hydrolyzed to yield glucose [52–55], which—together with the pentose-rich stream—can be used as a carbon source in the fermentation [52–54]. The fermentation for the conversion of the sugar fraction into itaconic acid can either be performed with the established *A. terreus* or with *Ustilago maydis*, a fungus growing with a yeast-like morphology [25, 56]. Both fermentation concepts were realized and evaluated. Depending on the fermentation, different downstream strategies can be applied to concentrate and purify itaconic acid. In this study, (in situ) filtration, (in situ) extraction, and crystallization were investigated and the influence of the preceding on the subsequent purification process was evaluated.

### OrganoCat pretreatment and enzymatic hydrolysis

**Fig. 2:**
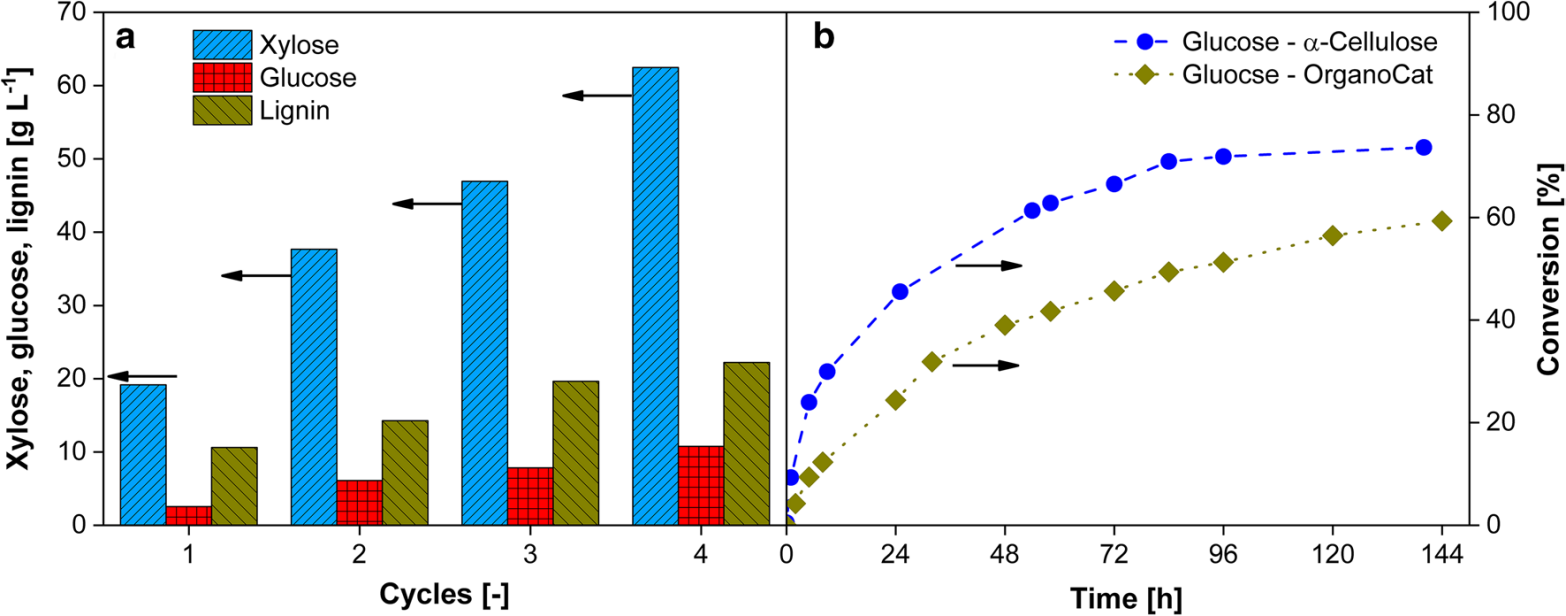
**a** Repeated-batch mode of the OrganoCat process by reusing the aqueous oxalic acid solution and the organic 2-MTHF solution [35]. Concentrations of xylose and glucose in the aqueous and lignin in the organic phase after chemo-catalytic pretreatment of beech wood with the OrganoCat process. Water and 2-MTHF were recycled four times. The cellulose pulp (51% (w/v)) was removed after each cycle for subsequent enzymatic hydrolysis. **b** Comparison of glucose concentration of hydrolysed 20 g L^−1^ α-cellulose and 20 g L^−1^ OrganoCat cellulose-rich beech wood pulp using Celluclast^®^ 500 μL g^−1^ substrate (enzyme loading of 32.5 FPU g^−1^) at 45 °C, 0.1 M sodium acetate buffer and a pH value of 4.8. Further details and information were published by Engel et al. [88] and Wang et al. [55]. The arrows refer to the relevant *y*-axis

To fractionate the mechanically pretreated raw material (dried 10 mm beech wood particles) into cellulose pulp, hydrolyzed hemicellulose sugars, and lignin, the so-called “OrganoCat technology” was used, which was developed by the Leitner group [35, 36]. This concept was developed from the well-established dilute-acid pretreatment called Organosolv process [57, 58], where an aqueous diluted acid (e.g., sulfuric acid) is used to hydrolyze part of the sugar polymers, while an organic solvent (e.g., ethanol, acetone) dissolves lignin. After the reaction, solid cellulose pulp is obtained and lignin is precipitated from the organic solvent syrup by dilution with water. OrganoCat uses a liquid/liquid two-phase reaction system in combination with a mild organic acid to affect the separation of the three main components of lignocellulose in a single processing step. In the original protocol, oxalic acid is used to hydrolyze selectively the non-cellulosic sugars—mainly xylose in case of beech wood—to be dissolved in the aqueous phase. The biogenic organic solvent 2-methyltetrahydrofuran (2-MTHF) is used as the second liquid phase, which extracts most of the liberated lignin from the reactive aqueous phase. The cellulose enriched fraction remains suspended as a solid pulp. The process itself was described in detail by vom Stein et al. [36] and Grande et al. [35]. Although already the non-optimized OrganoCat process was described as a competitive approach to other Organosolv-like processes [59], the biggest economic and ecological improvement can be achieved by an increased substrate to catalyst and solvent ratio via recycling of the liquid phases [35]. Figure 2a shows the results of the solvent recycling and the consequently increasing concentrations of sugars in the aqueous phase and of lignin in the organic phase. The cellulose pulp was removed after each cycle for further processing in the enzymatic hydrolysis step (Fig. 2b). In every cycle, 100 g L^−1^ of mechanically pretreated beech wood was processed. After recycling the liquid phases four times, lignin accumulates up to approx. 20 g L^−1^ in the organic phase. The organic phase containing the lignin can be further processed in a liquid/liquid extraction to transfer the lignin into an aqueous sodium hydroxide solution [42]. In the aqueous phase, the final concentrations of xylose and glucose were 65 g L^−1^ and 11 g L^−1^, respectively. This recycling significantly increases the economic balance of the OrganoCat process and is described in detail by Grande et al. [35].

Since the pretreatment significantly affects saccharification [37], enzymatic hydrolysis of the solid, cellulose-rich beech wood pulp was investigated (Fig. 2b). The hydrolysis of 20 g L^−1^ cellulose-rich beech wood pulp originating from OrganoCat pretreatment was compared to the hydrolysis of 20 g L^−1^ pure α-cellulose using a commercial cellulose cocktail, Celluclast^®^. After 144 h, 60% and 74% conversion into glucose was determined for cellulose-rich beech wood pulp and α-cellulose, respectively. The lower glucose conversion for the beech wood pulp can be explained by a different accessibility of the cellulose fibers for cellulases, caused by residual cellulose crystallinity and lignin content. However, adaption of the enzyme mixture of cellobiohydrolases, endoglucanases, and beta-glucosidase in the Celluclast^®^ enzyme cocktail to the specific cellulose structure of beech wood would further improve performance [55, 60–63].

### Lignin valorization

The utilization of lignin is essential for any biorefinery concept based on lignocellulose. A critical parameter for lignin processing is its high heterogeneity. Therefore, Stiefel et al. described a method based on statistical design of experiments to quantify the effect of temperature, alkalinity, catalyst, lignin concentration and current density on the molecular weight, monomer production, UV absorbance as well as acid solubility of the treated lignin [43]. For further lignin processing, different strategies were investigated on the engineering and on the bio-chemical level. A promising option is the usage of electrochemical membrane reactors to degrade lignin into low-molecular-weight compounds [38–41]. Alternative ways include chemical procedures like the ruthenium-catalyzed C–C bond cleavage [47], alcohol oxidation and subsequent cleavage into aromatics [44], copper- and vanadium-catalyzed oxidative cleavage [45], base-catalyzed depolymerisation [49] as well as mechanochemical degradation [46].

### Separation and recycling of oxalic acid

**Fig. 3:**
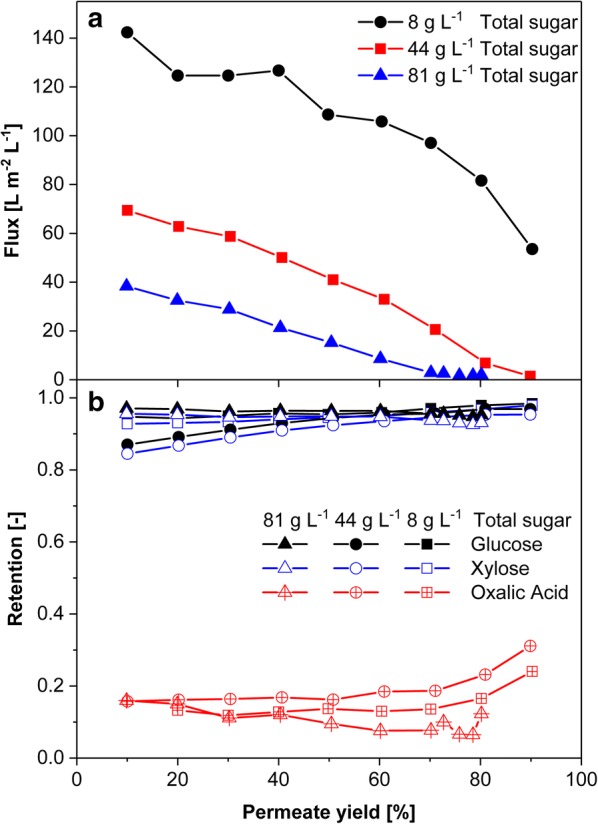
Nanofiltration for separation and recovery of oxalic acid from a sugar containing aqueous phase from the OrganoCat process (see also Fig. 1 and [36]). **a** Permeate flux in dependency of the permeate yield for different total concentrations. **b** Retention of glucose, xylose, and oxalic acid for different sugar concentrations. Conditions: Desal DL nanofiltration membrane in a stirred 1.4 lL dead-end filtration cell, 0.015 m^2^ membrane area, 40 bar, 300 rpm

As shown in Fig. 1, the mixture of oxalic acid, glucose, and xylose needs to be separated for further processing of the sugars and for recycling of oxalic acid. Membrane processes are a suitable approach for different separation tasks in biorefineries [64]. For the separation of oxalic acid from sugars, specifically, nanofiltration can be a valuable technology. In general, nanofiltration membranes separate by size and by charge. For the specific compound mixture of this study, glucose and xylose are retained, whereas oxalic acid permeates. The influence of varying sugar and oxalic acid concentrations was investigated, as both will vary, depending on the process conditions. The sugar concentration was investigated in a range of 6.1–61.3 g L^−1^ xylose with constant oxalic acid concentration of 11 g L^−1^. The initial total sugar concentration correlates negatively with the permeate flux (Fig. 3a). When the osmotic pressure by high sugar concentrations equaled the applied pressure, permeate flux drops to zero. Due to the higher osmolality, this point was reached at lower permeate yields with increasing initial total sugar concentration. As depicted in Fig. 3b, the applied Desal DL membrane retained glucose and xylose very well, while approximately, 20% oxalic acid was retained. A variation of oxalic acid concentrations in the range of 0.06–0.27 M, which covers the proposed oxalic acid concentration of 0.1 M [36], did not lead to significant changes of flux or retention (data not shown). As oxalic acid predominantly permeated through the membrane, there was a minor influence of oxalic acid on the osmotic pressure: between the minimum and maximum oxalic acid concentration the flux declined by less than 10%. In the covered concentration range, the pH of the solution varies between 1.25 and 2, where oxalic acid is either not charged or partially dissociated. No significant influence of electrostatic interaction was observed in the covered pH range (data not shown).

The results demonstrate that the proposed separation process is technically feasible. Recoveries for oxalic acid of 80% can be reached, depending on the initial sugar concentration. To process high monosaccharide concentrations, nanofiltration could be used in diafiltration mode. As a drawback, the oxalic acid concentration in the permeate will decrease. Meanwhile, it was also realized that part of the oxalic acid catalyst is decomposed upon extended operation times. Therefore, currently, experiments are conducted to find an acid which can easily be recycled and which is not decomposed under the fractionation conditions.

### Itaconic acid production with *A. terreus* and *U. maydis*

Industrial production of itaconic acid is commonly achieved with the filamentous fungus *A. terreus*, which exhibits high product yields and concentrations under optimal conditions [20]. Product formation in *A. terreus* is highly dependent on diverse factors including pH, oxygen supply, power input, phosphate concentration, and the presence of metal ions. In turn, product formation is poorly reproducible, if these factors are not precisely controlled [20, 25, 65]. Therefore, a second fungal producer, *U. maydis*, has been investigated by the TMFB consortium. Its wild type is a less efficient producer of itaconic acid than *A. terreus*, but its unicellular, non-filamentous morphology provides advantages for large-scale fermentation. Importantly, *U. maydis* is more tolerant to medium impurities. This is of lesser importance when working with pure glucose as feedstock, but crucial when using chemo-catalytically pretreated and hydrolyzed biomass. Besides itaconic acid, wild-type *U. maydis* produces several potentially interesting products like malic acid, succinic acid, and 2-hydroxyparaconic acid [56, 66–68]. The characterization of the metabolic pathway for itaconic acid [56] enabled rational metabolic engineering, resulting in the genetically modified *U. maydis* Δcyp3P_*etef*_ria1 in which itaconic acid production is increased, while by-product formation is reduced [69].

**Fig. 4:**
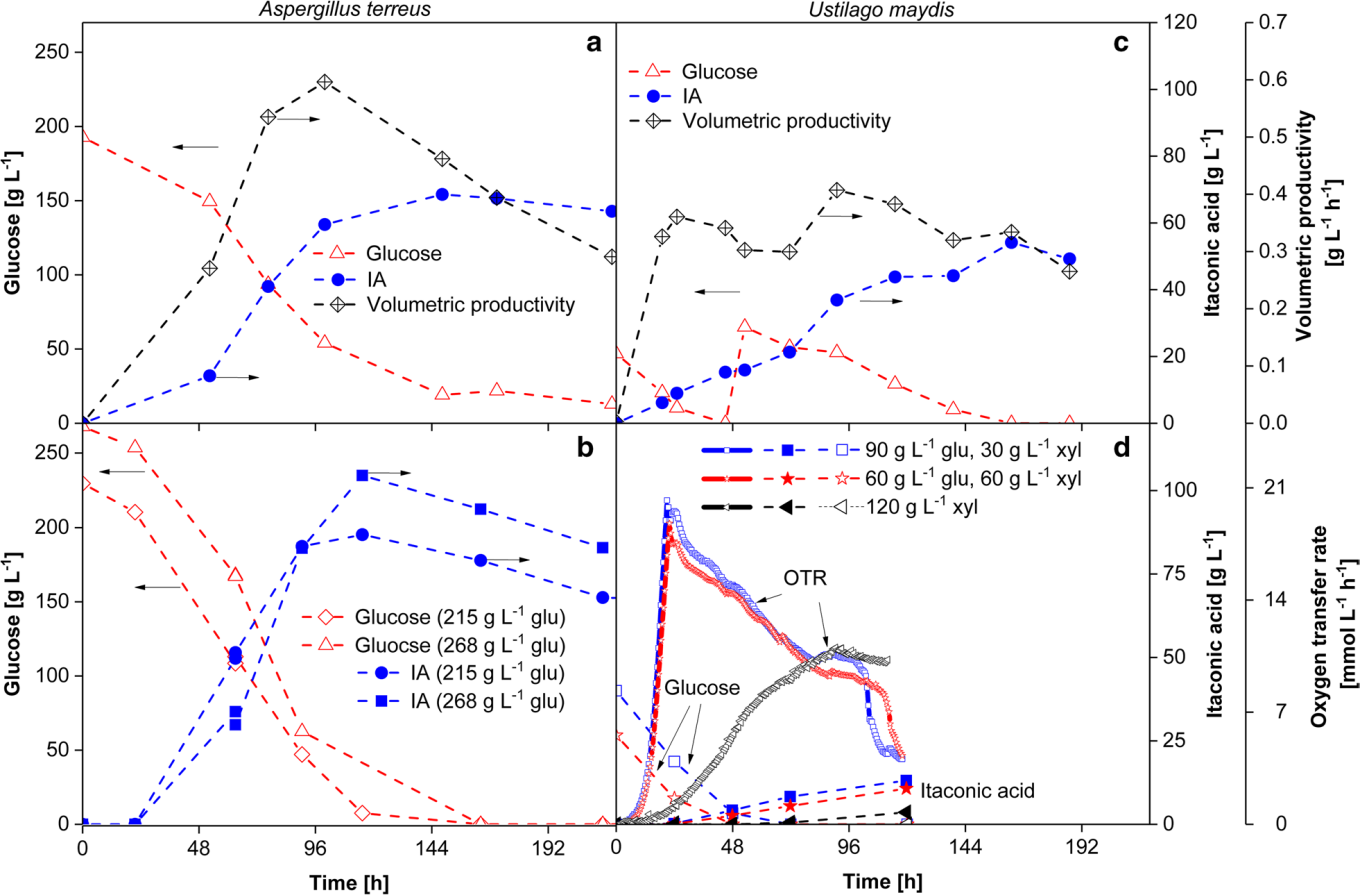
Comparison of itaconic acid formation by *A. terreus* (**a**, **b**) and *U. maydis* (**c**, **d**). **a** Batch fermentation of *A. terreus* with initial glucose concentration of 193 g L^−1^. **b** Batch fermentation of *A. terreus* with in situ itaconic acid extraction and initial glucose concentrations of 215 g L^−1^ and 268 g L^−1^. Further details were published by Kreyenschulte et al. [82]. **c** Batch fermentation of *U. maydis* with additional glucose pulse at 48 h. Further details were published by Geiser et al. [69]. **d** Substrate utilization and itaconic acid formation of *U. maydis* based on mixtures of glucose/xylose and pure xylose. Further details were published by Klement et al. [77]. The arrows refer to the relevant *y*-axis

Results for both organisms are presented in Fig. 4. The benchmark for the biotechnological production of itaconic acid is the batch cultivation with *A. terreus*. However, *A. terreus* volumetric productivities are rather low. Figure 4a represents a standard batch fermentation of *A. terreus* with a maximum itaconic acid concentration of 69 g L^−1^ and an initial substrate concentration of 193 g L^−1^ of pure glucose. The maximum volumetric productivity is 0.6 g L^−1^ h^−1^ after 100 h and an itaconic acid yield of 0.35 g_IA_ g_initial glucose_^−1^ was obtained. Significantly higher values for the final itaconic acid concentrations (up to 160 g L^−1^), the itaconic acid yield (0.58 g_IA_ g_consumed glucose_^−1^), and volumetric productivities up to 1.15 g L^−1^ h^−1^ have been published for different process conditions by several authors [24, 70]. Depending on the initial glucose concentration and the process mode, residual glucose between 20 g L^−1^ (as visible in Fig. 4a) up to 60 g L^−1^ [23, 24, 70] is left at the end of the fermentation. A detailed technoeconomic analysis would be necessary to determine the impact of the residual glucose concentration on the overall process and the necessity of a glucose recovery. It has to be mentioned that growth of *A. terreus* on the pretreated material was severely inhibited (data not shown). This is in agreement with reports on its high sensitivity towards impure substrates [20, 22, 71]. In addition to substrate impurities such as metal ions or lignin residues, the high concentrations of oxalic acid transferred from the OrganoCat process could, furthermore, have a negative impact on *A. terreus* in dependency of the pH value. A negative influence of residual 2-MTHF can presumably be excluded, since *A. terreus* was shown to tolerate medium saturated with this solvent [72]. Another important criterion for bio-based processes is met by *A. terreus* as it has the capability of converting C5 sugars [73, 74].

In Fig. 4c, a genetically modified *U. maydis* strain is shown. The maximum itaconic acid concentration is approx. 55 g L^−1^ after 163 h and a maximum volumetric productivity of 0.4 g L^−1^ h^−1^ after 90 h is achieved. The product yield is 0.48 g_IA_ g_initial_ _glucose_^−1^. *U. maydis* is known for converting hemicellulose into fungal biomass and itaconic acid, as shown in Fig. 4d. As represented by the oxygen transfer rate (OTR) and itaconic acid concentration, *U. maydis* is not only capable of growing and producing itaconic acid on pure glucose/xylose mixtures, but also on pure xylose. It can also degrade xylan and can be engineered to secrete xylanases and cellulases, making it potentially applicable in a consolidated bioprocess [75, 76]. In general, organisms which are capable to convert C5 and C6 sugars are preferable for biorefinery processes. To investigate the robustness of *U. maydis* and its capability to convert also hemi-cellulosic sugars from pretreated beech wood, investigations were performed by Klement et al. [77]. It could clearly be shown that *U. maydis* tolerates oxalic acid concentrations up to 0.1 M, which is the applied concentration in the OrganoCat process, presented above (Fig. 2a). The oxalic acid concentration transferred into the fermentation step depends on the operation conditions of the nanofiltration, as presented in Fig. 4. Further experiments investigating the robustness of *U. maydis* are presented in Klement et al. [77].

To increase the volumetric productivity, a continuous process mode is an option. However, product concentrations are often lower in continuous processes compared to repeated-batch and fed-batch processes. In addition, the biocatalyst is also continuously washed out. For this reason, Carstensen et al. [78] used an in situ membrane module in a continuously operated stirred tank reactor to increase the volumetric activity and to generate a cell free, itaconic acid containing permeate stream. A volumetric productivity of 0.8 g L^−1^ h^−1^ was obtained in that work, even though only an unoptimized wild-type strain *U. maydis* MB215 was used in these experiments. Subsequently, an advanced reversed flow diafiltration technique was developed [79, 80]. A combination of genetically enhanced strains of *U. maydis* and the membrane bioreactor promises even higher volumetric productivities.

### Extraction and back extraction of itaconic acid

The comparison of different downstream opportunities (crystallization, reactive extraction, precipitation, electrodialysis, diafiltration, and adsorption) for itaconic acid was published by Magalhães et al. [81] and reflects also the experience of this study that reactive extraction is superior in respect to energy demand and scalability. Therefore, an in situ removal by reactive extraction has been realized. In Fig. 4b, results of experiments with isopropyl myristate as organic carrier solvent and trioctylamine as reactant for an in situ itaconic acid reactive extraction are depicted. For both initial concentrations, 215 g L^−1^ as well as 268 g L^−1^ glucose is completely converted into itaconic acid. These results reflect the biocompatibility of the solvent fraction to *A. terreus*, which is also important if process water is recycled. Remarkably, no glucose remains in the medium at all, even though higher initial glucose concentrations were added than in the experiment, as shown in Fig. 4a. The maximum itaconic acid concentrations are 87 g L^−1^ and 105 g L^−1^ with a yield for both experimental conditions of approx. 0.41 g_IA_ g_initial glucose_^−1^. Therefore, in situ extraction of itaconic acid is a powerful tool to increase the efficiency of the process [82, 83]. Further details are described in Kreyenschulte et al. [82].

**Fig. 5:**
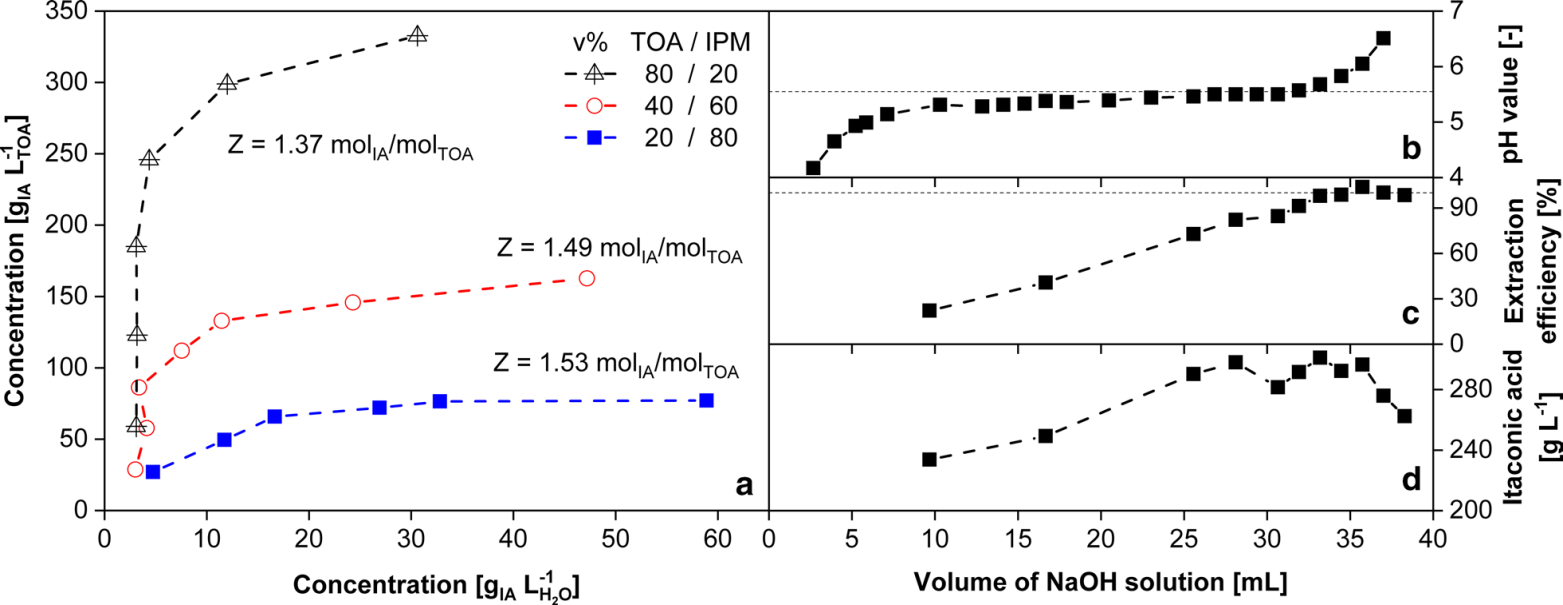
**a** Reactive extraction of itaconic acid with different mixtures of isopropyl myristate (IPM) and trioctylamine (TOA). Conditions: contact time per measurement 2 h, *T* = 25 °C. **b**–**d** Back extraction of itaconic acid based on **b** pH-shift by adding NaOH, **c** its efficiency, and **d** final concentration. Further details and information were published by Kreyenschulte et al. [82]

Alternatively, extraction of itaconic acid can also be performed in an external process unit. As depicted in Fig. 5, 3 g L^−1^ itaconic acid is the minimal concentration for extraction from the aqueous to the organic phase without additional acidification. Since only protonated acid can be extracted by trioctylamine, the lower boundary is based on the pH-dependent protonation–dissociation equilibrium around the pK_a1_ value of 3.55. In dependency of the volumetric ratio between the reactant trioctylamine and organic phase isopropyl myristate, the maximum itaconic acid amount increases in the organic phase with the added amount of trioctylamine. Based on the presented data, a loading factor Z, defined as the molar ratio between itaconic acid and the reactive component trioctylamine, can be calculated. As shown in Fig. 5a, the loading factor Z for this extraction system is in a small range between 1.37 and 1.53 mol_IA_ mol_TOA_^−1^ [84, 85]. Consequently, the loading factor Z hardly depends on the ratio between trioctylamine and isopropyl myristate. With respect to fermentations presented for *A. terreus* (Fig. 5a) and *U. maydis* (Fig. 5c) containing 60 g L^−1^ itaconic acid, an enrichment of factor 5 (up to 300 g L^−1^) is, therefore, feasible within the organic phase.

For the back extraction of itaconic acid into the aqueous phase (Fig. 1), a pH shift by the addition of alkaline solution of, e.g., NaOH, can be realized. With pH values in the aqueous phase higher than 5.55 (pK_a2_), back extraction with an efficiency of 100% is feasible (Fig. 5b, c). The consequence is the formation of a salt. By adding approx. 35 mL NaOH per liter isopropyl myristate containing 300 g L^−1^ pure itaconic acid, the product can be completely back extracted into the water phase. The efficiency of this back extraction can be described by the molar ratio of 0.69 mol_IA_ mol_NaOH_^−1^.

#### Crystallization of itaconic acid

**Fig. 6:**
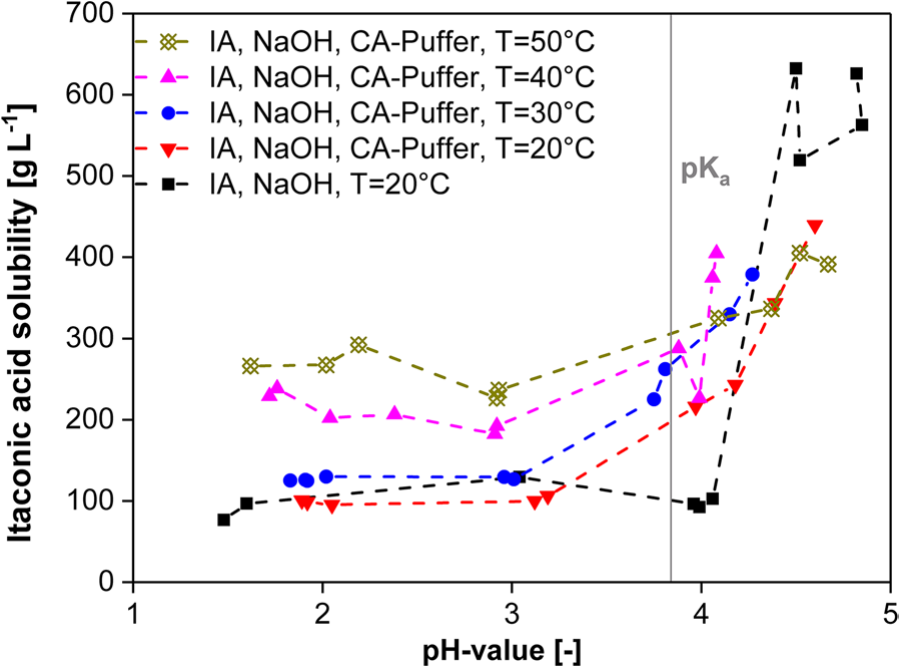
Operation of pH-shift crystallization. Solubility of itaconic acid (IA) as a function of pH value as well as in presence of citric acid (CA) buffer as a function of pH value and temperature, determined by excess method and HPLC analysis. The vertical line marks the lower pK_a1_ value of itaconic acid at pH 3.84. The pH was readjusted after 2 h by the addition of (45 wt%) sodium hydroxide solution

To gain pure, solid itaconic acid, the aqueous solution obtained by back extraction can be fed into a pH-shift crystallization unit (Fig. 1). Results of the experiment are shown in Fig. 6. The itaconic acid solubility in the aqueous solution increases drastically around the pK_a1_ value (3.84), as previously shown for succinic acid [86]. As fermentations especially with *U. maydis* need pH control, the addition of base is necessary. Therefore, salt or buffer concentration in the liquids transferred to the following unit operations after the final separation (reactive extraction) can be calculated based on the weight and flow measurement through the base pump. The tested buffer solution consisting of citric acid and sodium chloride shows a decrease of the steep increase of itaconic acid crystallization to only fourfold around the pK_a1_ (*T* = 20 °C) and decreases even further with higher temperatures (*T* = 50 °C). pH-shift crystallization should be operated at low temperatures [21], which reduces the solubility of itaconic acid below a pH of 3.84 drastically.

#### Economic background

The published industrial benchmark for itaconic acid production is a batch process based on molasses. By beginning of 2018, the average price for molasses in Europa was approx. 128 € t^−1^ (http://www.proplanta.de). Due to changes in sugar market regulation by the European government, the price of sugar dropped to approx. 110 € t^−1^ in August 2018 (New York Mercantile Exchange). Taking into account that molasses contains only 43–45% of convertible sugars, the often-mentioned industrial process loses its relevance as benchmark under the current situation on the sugar market—at least in Europa. Nieder-Heitmann et al. published different economic scenarios for itaconic acid producing biorefinery concepts [87]. Among others, the authors have compared itaconic acid production based on glucose with production based on lignocellulose, which should theoretically be more price competitive. In case of a lignocellulosic feedstock, most economically relevant parameters of the process are the itaconic acid yields based on glucose as well as on xylose, followed by the volumetric productivity and the initial glucose concentration (which reflects the efficiency of pretreatment and enzymatic hydrolysis). With respect to the itaconic acid yield on glucose, both compared organisms *U. maydis* and *A. terreus* are in the same range of approx. 0.5 g_IA_ g_initial_ _glucose_^−1^ (theoretical yield 0.72 g_IA_ g_glucose_^−1^, [19]) depending on experimental conditions and cited publications. *A. terreus* as well as *U. maydis* can (co-)metabolize xylose into biomass and itaconic acid in low yields and rates. The published volumetric productivity of *A. terreus* is higher, but *U. maydis* seems to have a lot of potential for further improvement on the genetic level as well as on the process engineering level, due to its higher growth rate, its resistance towards impurities, and its single-cell morphology. Since the impact of the initial glucose concentration is significantly lower in comparison to the itaconic acid yields [87], it might be tolerable if some glucose remains in the fermentation broth as long as high product yields can be achieved and the downstream processing is not severely affected. However, to quantify the impact of all process relevant and economic parameters in detail, a technoeconomic analysis would be essential.

## Conclusion

A comprehensive examination of the itaconic acid process and the interfaces between the unit operations demonstrates the impact of realistic process conditions on the feasibility, product yields, choice of microorganisms, and operation mode. The decision for a suitable chemo-catalytic fractionation method is probably the most essential choice to make. As shown by the results of the cellulose hydrolysis as well as fermentation with *U. maydis* or *A. terreus*, all the following steps will be influenced by the digestibility of the pretreated biomass by the cellulolytic enzymes and inhibitors originating from the pretreated biomass. For the microbial conversion of pretreated biomass, evaluation parameters like robustness against impurities and inhibitors and flexibility of feedstock are at least as important as classical parameters like yield and volumetric productivity. In this context, the genetically enhanced *U. maydis* is ultimately superior to *A. terreus*. In case of itaconic acid and the achieved concentrations after fermentation, reactive extraction by trioctylamine and isopropyl myristate is a suitable way to significantly increase the itaconic acid concentration before crystallization. Experiments with in situ extraction demonstrate the biocompatibility of trioctylamine and isopropyl myristate. Therefore, a recycling of the aqueous phase containing dissolved extractant would be harmless for the microbial system. The presented results towards an integrated itaconic acid process clearly demonstrates that the shift to lignocellulosic substrates challenges existing processes and shows the importance of including the interfaces between unit operations into the research efforts.
